# Puerperal Convulsions with Albuminuria1Reported to the Section in Obstetrics and Gynecology, Buffalo Academy of Medicine, April 24, 1894.

**Published:** 1894-07

**Authors:** Benj. G. Long

**Affiliations:** 520 Elmwood Avenue; Buffalo


### PUERPERAL CONVULSIONS WITH ALBUMINURIA.[1]

By BENJ. G. LONG, M. D., Buffalo.

Mrs. T., American, aged 30, married ; seen first June 15, 1892. Pale and waxy in appearance ; face puffy ; feet and limbs very edematous ; coughing almost persistently, headache, sleeplessness, and some impairment of vision ; reports that she is about eight months pregnant ; second pregnancy ; first child born dead, labor

1. Reported to the Section in Obstetrics and Gynecology, Buffalo Academy of Medicine, April 24, 1894.

normal. Family history good. Physical examination shows moist rales throughout lung and some edema. Examination of urine showed specific gravity 1010, no sugar, albumin so abundant that, upon boiling, the urine became so solid that it would not run from the test-tube when inverted. Hyaline and granular casts abundant. By the use of cathartics, diuretics, hot baths, and milk diet, the amount of urine was increased, the albumin diminished, as was also the edema of extremities and lungs, with consequent improvement of the cough and the wellbeing of the patient generally. On the evening of July 4, 1892, she was delivered of a healthy child, the labor being normal, and chloroform administered. After the birth of the child, the patient seemed less bright than before, but there were no convulsions. The next day, July 5th, the patient was not quite clear in her head, but seemed to be somewhat improved mentally, had passed her water freely, which, upon examination, showed much albumin and also casts.

The second day after delivery, July 6th, I was summoned early in the morning, on account of her having had a convulsion. I found her comatose, breathing stertorously, face livid, lips and nails blue, and with a rapid, full pulse. My brother, Dr. Eli H. Long, now saw her with me. By the use of full doses of chloral, croton oil and the steam bath, further convulsions were prevented, but the condition of the patient was so unpromising that an unfavorable prognosis was made. The condition of the patient gradually improved, however, but her mind was clouded for at least two weeks. She was soon up and about, and after a couple of months resumed her avocation of conducting a stall for baked goods on the market. The albumin in her urine diminished and she soon appeared as well and hearty as ever.

I warned her husband and herself of the danger which would be incurred in the event of her becoming pregnant again, and cautioned her to be careful of exposure to wet and cold, etc.

About September 1, 1893, I was informed by Mr. T. that his wife was again pregnant and about three months advanced. I obtained a sample of urine for examination, and found it normal in every particular. Frequent tests, at regular intervals, showed the urine always to be normal. The patient was delivered of her third child, February 27, 1894, by a precipitous labor, the child being born upon my arrival at the house. The child was healthy, but small, and the mother made an excellent recovery, with no untoward symptoms. Frequent urinary analyses after labor showed the urine to be normal, save on the second day only a trace of albumin was found.

520 Elmwood Avenue.

